# Invasive Primary Melanoma of the Kidney: A Rare Presentation of an Uncommon Malignancy and Review of Literature

**DOI:** 10.1155/criu/3596493

**Published:** 2026-07-22

**Authors:** Joshua Ghoulian, Jersey-Kate Castillo, Ronaldo Dario Gnass, Vicente Ramos-Santillan, Antoin Douglawi, David Caba, Muhannad Alsyouf

**Affiliations:** ^1^ Department of Urology, Loma Linda University Health, Loma Linda, California, USA, lluh.org; ^2^ Division of Urology, Riverside University Health, Moreno Valley, California, USA; ^3^ Division of Pathology, Riverside University Health, Moreno Valley, California, USA; ^4^ Division of Surgical Oncology, Riverside University Health, Moreno Valley, California, USA

**Keywords:** kidney cancer, melanoma, nephrectomy

## Abstract

Primary melanoma is a very rare malignancy arising from the kidney. We present a case of a 78‐year‐old female who presented with locally advanced primary kidney melanoma requiring complex surgical intervention and multidisciplinary management. The patient underwent an open left radical nephrectomy with distal pancreatectomy, splenectomy, and colonic resection, followed by adjuvant immunotherapy. We additionally review the literature regarding the diagnosis and management of primary renal melanoma.

## 1. Introduction

Renal malignancies accounted for an estimated 434,840 new cases and 155,953 deaths worldwide in 2022, with clear cell renal cell carcinoma accounting for approximately 75% of cases [1]. The remaining 25% consist of less common histologic subtypes, including papillary, chromophobe, collecting duct, and medullary carcinomas. Additional rare histologies, which represent approximately 4% of kidney malignancies, pose a clinical challenge due to their rarity and the lack of well‐defined management algorithms [1]. Localized rare renal malignancies are typically managed with surgical resection, whereas metastatic disease is treated with systemic therapy, including immune checkpoint inhibitors, targeted agents against molecular pathways, or chemotherapy [2, 3].

One such rare entity includes primary renal melanoma, with only seven instances documented in the literature [4–10]. This malignancy is referred to as mucosal melanoma (MML), which is a rare subtype that accounts for 1.3% of all melanomas and originates in the non‐UV‐exposed areas such as the mucosal lining of the head and neck, anorecta, and genitourinary tract [11]. All these descriptions have reported on the management of locally noninvasive tumors. Given the rarity of this diagnosis, clinicians must often rely on individual case reports and small case series to guide diagnosis and treatment decisions. To date, there has been no description, to our knowledge, of locally advanced primary melanoma of the kidney with invasion into surrounding structures requiring complex surgical treatment and multidisciplinary management.

We describe a case of a 78‐year‐old female with a large 16‐cm primary renal MML invading into the pancreas, colon, and spleen, which was managed with open left radical nephrectomy and en bloc left colectomy, subtotal pancreatectomy, and splenectomy. Details regarding the surgical technique are contained in this report.

## 2. Case Presentation

A 78‐year‐old female with a past medical history of poorly controlled asthma and hypertension presented to the emergency department after a recent fall with progressive left‐sided abdominal pain and abdominal fullness over several weeks. She denied hematuria, weight loss, or constitutional symptoms. On physical examination, a large, firm, nontender mass was palpable along the left flank, extending from the subcostal margin to the anterior superior iliac spine. Laboratory evaluation showed normal renal function and hemoglobin levels, without evidence of metabolic abnormalities. Computed tomography (CT) of the chest, abdomen, and pelvis revealed a 16 cm heterogeneously enhancing left renal mass concerning for malignancy, without distant metastasis. The lesion demonstrated radiographic invasion into the pancreatic tail, splenic hilum, and adjacent large bowel mesentery, with displacement of the great vessels. After multidisciplinary tumor board review and extensive discussion with the patient regarding management options, including preoperative biopsy versus upfront surgical management, she elected to proceed with definitive surgical resection in collaboration with the surgical oncology team. Details of the operative approach and postoperative course are presented below.

### 2.1. Surgical Management

The patient underwent an open left radical nephrectomy with retroperitoneal lymph node dissection, en bloc colectomy, subtotal pancreatectomy, and splenectomy. Through a midline abdominal incision, a large left renal mass was identified with invasion of the descending colon mesentery extending from below the splenic flexure to above the sigmoid colon (Figure 1). The mass demonstrated close adherence to the aorta and left common iliac artery, requiring careful vascular dissection using the “split‐and‐roll” technique. This approach facilitates circumferential identification and preservation of major vessels through dissection along the adventitial plane.

**Figure 1:**
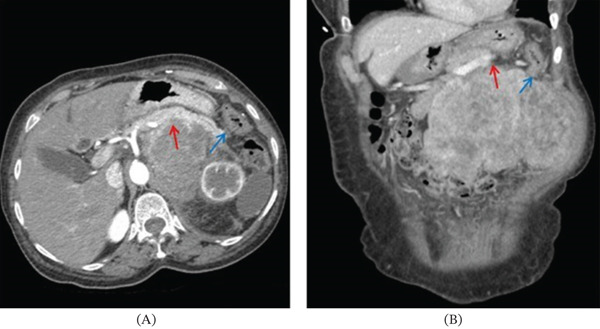
(A) Axial and (B) coronal computed tomography scan displaying tumor invasion of the pancreas (red arrow) and tumor invasion into the bowel (blue arrow).

The tumor involved the inferior mesenteric artery, which was ligated at its origin, and extended superiorly to invade the splenic vein and distal pancreas (Figure 2A). In collaboration with the surgical oncology service, which managed the pancreatic, splenic, and colonic components of the operation, an en bloc resection was performed, including the left kidney, descending colon, distal pancreas, and spleen, to ensure negative margins. The pancreas and spleen were removed in continuity following vascular control and dissection of the short gastric vessels.

**Figure 2:**
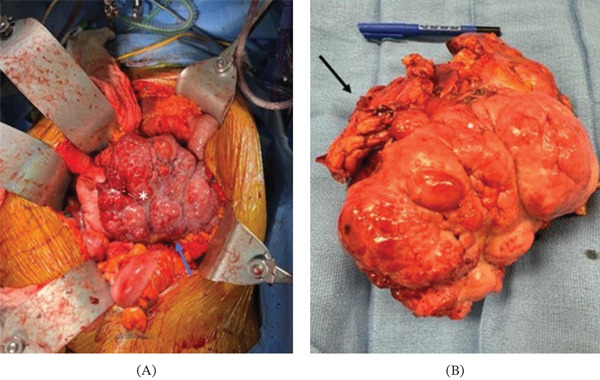
(A) Retroperitoneal renal mass (asterisk) with invasion of the transverse and sigmoid colon mesentery (blue arrow). (B) Retroperitoneal renal mass (asterisk) with pancreatic staple line (black arrow).

Colonic reconstruction was performed with a side‐to‐side colo‐colostomy after confirming adequate perfusion using indocyanine green fluorescence imaging. A diverting loop ileostomy was created to protect the anastomosis. The specimen, consisting of the left kidney and associated en bloc resection, was removed intact (Figure 2B). Drains were placed along the pancreatic and retroperitoneal beds.

The patient′s postoperative course was notable for delayed return of bowel function but was otherwise without major complications. She was discharged on postoperative day 21 with preserved renal function (serum creatinine 0.55 mg/dL).

### 2.2. Pathology

Pathologic examination of the left kidney revealed a 16.2‐cm melanoma. Histologic evaluation with H&E staining demonstrated tumor lobules separated by fibrous septa (Figure 3). Immunohistochemistry was positive for vimentin, supporting mesenchymal differentiation, and negative for epithelial and hematopoietic markers (AE1/AE3, CD45, CD20, CD3, CD15, and CD30), helping to exclude carcinoma and lymphoma. Additional melanocytic markers, including HMB‐45 and S‐100, were also positive (if tested; otherwise omit). Molecular profiling using CancerTYPE ID and next‐generation sequencing identified a PTEN mutation and 100% PD‐L1 expression, whereas BRAF, KIT, and NRAS were negative. The tumor was microsatellite stable (MSS), and pan‐TRK was not expressed. These findings support the diagnosis of primary renal melanoma and provide insight into potential therapeutic considerations, including suitability for immunotherapy.

**Figure 3:**
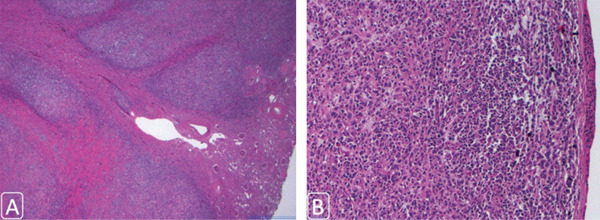
(A) Panoramic view of tumor with tumor lobules surrounded by fibrous septa with H&E staining at 12.5X magnification. (B) High magnification of pleomorphic, large neoplastic cells.

### 2.3. Multidisciplinary Treatment Planning

Following final pathologic diagnosis, the case was reviewed at a multidisciplinary tumor board involving urology, surgical oncology, pathology, radiology, and medical oncology. Given the aggressive nature of MML and the high risk of recurrence despite complete surgical resection, adjuvant systemic therapy was recommended. After discussion of treatment options, the patient was initiated on adjuvant nivolumab. This management strategy was extrapolated from contemporary data in cutaneous melanoma supporting the use of anti‐programmed death‐1 (PD‐1) immune checkpoint inhibitors, including nivolumab and pembrolizumab, in patients with high‐risk resected disease [12]. At 11 months of follow‐up, the patient remains without evidence of disease recurrence on surveillance imaging.

## 3. Discussion

Melanoma is the 17th most common cancer globally, with an estimated 331,700 new cases diagnosed in 2022 [13]. MML is diagnosed at a median age of 67 years, occurs more often in females, and presents 12 years earlier than cutaneous melanoma, which predominantly affects males [11]. Renal melanoma more commonly presents as a metastatic lesion to the kidney although most cases are in the setting of already widespread disease and isolated renal disease is uncommon [14]. MML specifically is a rare and aggressive subtype of melanoma with distinct metastatic spread patterns and poor survival outcomes (5‐year survival rates of 16%–27%) [15]. Primary renal melanoma is a form of MML that arises from melanocytes within the urothelial mucosa of the renal pelvis or collecting system and not the renal parenchyma [11]. To our knowledge, this represents the eighth reported case of primary renal melanoma in the literature and, to our knowledge, the only case characterized by a very late presentation of locally advanced disease. The differential diagnosis includes retroperitoneal melanoma with invasion into the kidney versus an alternative primary tumor that regressed; however, neither has been confirmed to date.

Notable factors for diagnosing primary MML include a thorough medical history, physical examination, diagnostic evaluation with imaging, and tumor identification with immunohistochemistry. A key factor to the diagnosis of primary renal melanoma is confirming the absence of another primary lesion and potential for metastatic disease to the kidney rather than primary involvement [10, 11, 16]. Tumor identification can be completed with immunohistochemistry positivity towards HMB45, S‐100 protein, actin, and vimentin in addition to genomic testing of NF1 and RAS mutations [17]. In our case, vimentin was positive by immunohistochemistry, and a gene expression assay, which predicted tumor origin in diagnostically challenging cases, showed an 89% probability of melanoma as the primary site [18].

As demonstrated in Table 1, management of primary renal melanoma in the literature consistently started with definitive surgical removal. Primary tumors ranged from 2.2 to 7 cm in prior cases, whereas our patient′s tumor measured 16 cm in its largest dimension [4–10]. Across the prior cases, surgical management included nephroureterectomy, radical nephrectomy, and regional lymphadenectomy. Due to late presentation in this case, the renal mass was initially identified with direct invasion into the pancreas, colon, and spleen, necessitating multidisciplinary surgical management and multiorgan resection.

**Table 1:** Clinical and histopathologic features of primary renal mucosal melanoma reported to date.

	Frasier et al. [5]	Fujimoto et al. [10]	Tajima et al. [7]	Bayazit et al. [8]	Tasdemir et al. [9]	Liapis et al. [6]	Onogi et al. [4]	Present case
Demographics								
Age (years)	37	54	74	37	67	35	45	78
Sex	Male	Female	Female	Male	Male	Male	Female	Female

Tumor and investigations								
Tumor (largest dimension)	5	2.5	3.5	8	4.5	2.5	7	16.2
Tumor location	Right renal pelvis	Left kidney: lower pole	Right kidney: upper pole	Right medial lobe of kidney	Right kidney: lower pole	Right renal pelvis	Right kidney: lower pole	Left kidney
Invaded organs	No evidence	No evidence	No evidence	Lungs and heart (kidney adjacent fatty tissue invasions)	No evidence	Liver (Segment VII) and spleen at 6‐month follow‐up	No evidence	Pancreas, colon, and spleen

Surgical procedures								
Operative approach	Nephroureterectomy	Partial resection of renal tumor	Right radical nephrectomy	Radical nephrectomy, adrenalectomy, and paracaval and interaortacaval lymph node dissection	Right radical nephrectomy	Right nephrectomy and liver biopsy	Laparoscopic right nephrectomy	Open left radical nephrectomy + en bloc left colectomy, subtotal pancreatectomy, splenectomy, and ileotomy creation

Pathological findings								
Histology	Organized nests, markedly pleomorphic cells with large amounts of eosinophilic cytoplasm and large hyperchromatic nuclei, scatterings of “golden, dust‐like pigment”	Sheets of brown pigmented cells with occasional mitotic figures, nuclei are bizarre, nuclear atypia, and nucleolar are scant	Extensive proliferation of primarily clear cells in the cytoplasm	Epithelioid cell tumor in nodular architecture, prominent melanin pigmentation in stroma and cytoplasm	Abundant eosinophilic cytoplasm with enlarged nuclei; tumor thrombus in renal vein lumen	Marked heterogeneity displaying predominant nest of clear cells with microvacuolated cytoplasm and round nuclei; also nests of tumor cells around urothelium; melanin pigment deposition present	Well‐encapsulated tumor with papillary growth of spongiotic/eosinophilic cytoplasm and prominent nucleoli	Undifferentiated malignant round cell neoplasm, undetermined origin
Immunohistochemistry	(+): S‐100 protein, and stains for melanoma antigens. (−): iron and keratine stains	(+): HMB‐45. (−): S‐100 protein, cytokeratin, chromogranin, and epithelial membrane antigen	(+): Fontana–Masson stain, vimentin, S‐100 protein, neuron‐specific enolase, and HMB‐45	(+): HMB‐45 and Fontana–Masson stain	(+): HMB‐45 and S‐100 protein	(+): HMB‐45, S‐100, Melan‐A, WT‐1, CD10, and vimentin. (−): Pan‐cytokeratin, SMA, chromogranin, synaptophysin, and CD56	(+): Melan‐A, HMB‐45, and S‐100. (−): TFE3, TFEB, CK7, CA9, and AE1/AE3	(+): Vimentin. (−): AE1/AE3, CD45, CD 20, CD3, CD15, CD30, and RCC

Postsurgical course								
Post‐op treatment	Bacuìillius Calmette–Guerin and allogenic melanoma cell vaccination	Chemotherapy	Human lymphfoblastoidinterferon‐alfa therapy (1 month)	Fotemustine and interferon‐alfa 2b therapy (3 months)	Human lymphfoblastoidinterferon‐alfa therapy (1 month)	Chemotherapy	None	Nivolumab
Follow‐up	22 months	44 months	27 months	12 months	2 weeks	6 months	18 months	10 months to date

Because of the rarity of primary renal melanoma, no disease‐specific treatment guidelines currently exist. Consequently, management strategies are largely extrapolated from evidence derived from cutaneous and other MML subtypes. Surgical resection remains the cornerstone of treatment for localized disease, whereas immune checkpoint inhibitors targeting the PD‐1 pathway have become standard components of systemic therapy for patients at high risk of recurrence. Contemporary guidelines support the use of adjuvant nivolumab or pembrolizumab following complete resection of high‐risk melanoma, and these principles have increasingly been applied to rare primary renal melanoma cases despite the absence of prospective disease‐specific data [12].

In conclusion, primary renal melanoma remains an exceptionally rare and diagnostically challenging entity within the spectrum of rare kidney malignancies. Our case adds to the limited body of literature by presenting the largest reported tumor to date and the only instance characterized by locally advanced disease requiring multidisciplinary surgical intervention. This highlights the importance of thorough diagnostic workup and preoperative planning, including imaging, immunohistochemistry, and genomic testing, to differentiate primary lesions from metastatic disease. There is potential value in applying treatment principles from cutaneous melanoma to this rare variant, though more research is needed to inform standardized management. Current recommendations from the NCCN guidelines recommend surgical resection for localized disease with adjuvant or neoadjuvant systemic therapy with immune checkpoint inhibitors versus targeted therapies [19].

## Funding

No funding was received for this manuscript.

## Conflicts of Interest

The authors declare no conflicts of interest.

## Data Availability

Research data are not shared.
